# New Light on Chloroplast Redox Regulation: Molecular Mechanism of Protein Thiol Oxidation

**DOI:** 10.3389/fpls.2019.01534

**Published:** 2019-11-22

**Authors:** Keisuke Yoshida, Yuichi Yokochi, Toru Hisabori

**Affiliations:** Laboratory for Chemistry and Life Science, Institute of Innovative Research, Tokyo Institute of Technology, Yokohama, Japan

**Keywords:** chloroplast, 2-Cys peroxiredoxin, hydrogen peroxide, oxidation, redox regulation, thioredoxin, thioredoxin-like2

## Abstract

Thiol-based redox regulation is a posttranslational protein modification that plays a key role in adjusting chloroplast functions in response to changing light conditions. Redox-sensitive target proteins are reduced upon illumination, which turns on (or off in a certain case) their enzyme activities. A redox cascade *via* ferredoxin, ferredoxin-thioredoxin reductase, and thioredoxin has been classically recognized as the key system for transmitting the light-induced reductive signal to target proteins. By contrast, the molecular mechanism underlying target protein oxidation, which is observed during light to dark transitions, remains undetermined over the past several decades. Recently, the factors and pathways for protein thiol oxidation in chloroplasts have been reported, finally shedding light on this long-standing issue. We identified thioredoxin-like2 as one of the protein-oxidation factors in chloroplasts. This protein is characterized by its higher redox potential than that of canonical thioredoxin, that is more favorable for target protein oxidation. Furthermore, 2-Cys peroxiredoxin and hydrogen peroxide are also involved in the overall protein-oxidation machinery. Here we summarize the newly uncovered “dark side” of chloroplast redox regulation, giving an insight into how plants rest their photosynthetic activity at night.

## Introduction

Sunlight is the primary energy for photosynthesis, but is also the most variable environmental cue in plant habitats. It is thus critical for plant growth and maintenance to regulate photosynthesis in flexible and suitable ways. In fact, as the site for photosynthesis, chloroplasts have acquired multiple adaptive strategies to light. Accumulating research has expanded our understanding of how chloroplasts regulate their own physiology under continuously fluctuating light environments (Niyogi, 2000; Takahashi and Badger, 2011; Yamori, 2016), but further studies are needed for a better understanding.

Thiol-based redox regulation is one of the posttranslational protein modifications. The most well-known example is the dithiol/disulfide interconversion of a redox-active Cys pair. A key factor for the redox regulation is thioredoxin (Trx), a small soluble protein first discovered in *Escherichia coli* and now known to occur in all organisms (Laurent et al., 1964; Holmgren, 1985; Jacquot et al., 1997). Trx has a conserved amino acid sequence of WCGPC at its active site. Using a Cys pair in this motif, Trx mediates the dithiol/disulfide exchange reaction with its target proteins and thereby transmits reducing power to the targets. In addition to regulating the enzyme activity, Trx-derived reducing power can be used for the catalytic cycle itself by some proteins (*e.g.*, peroxiredoxin (Prx), Dietz, 2011).

As well as other organisms and subcellular compartments, plant chloroplasts commonly have a redox-regulatory system; however, its mode of action is critically different from that of others. Namely, chloroplast redox regulation is coupled with light. The electron transport chain (ETC) in the thylakoid membrane converts absorbed light energy into reducing power, a part of which drives the redox-regulatory system in chloroplasts. In this system, reducing power pooled by ferredoxin (Fd), a small [2Fe-2S] electron carrier protein (Arnon, 1988), is transferred to Trx *via* Fd-Trx reductase (FTR). FTR is a [4Fe-4S] cluster-containing heterodimeric protein that serves as the signaling hub to link the ETC to Trx (Dai et al., 2004). Trx in the reduced state, in turn, provides reducing power to the redox-sensitive target proteins in chloroplasts. As the example of redox-regulated proteins in chloroplasts, four Calvin-Benson cycle (CBC) enzymes (glyceraldehyde-3-phosphate dehydrogenase (GAPDH), fructose-1,6-bisphosphatase (FBPase), sedoheptulose-1,7-bisphosphatase (SBPase), and phosphoribulokinase (PRK)) are classically known (Michelet et al., 2013). Because these enzymes are activated upon reduction, a redox cascade composed by Fd, FTR, and Trx makes it possible to turn on CBC in a light-dependent manner (Buchanan, 1980; Buchanan et al., 2002). Our understanding of CBC redox regulation is even now growing. For example, its molecular details and evolutionary traits have been further elucidated by recent structural studies (FBPase and SBPase from *Physcomitrella patens*, Gütle et al., 2016; PRK from *Chlamydononas reinhardtii* and *Arabidopsis thaliana*, Gurrieri et al., 2019).

The Fd/FTR/Trx-based regulatory pathway was discovered by Buchanan and colleagues in 1970s (Buchanan et al., 1967; Buchanan et al., 1971; Buchanan and Wolosiuk, 1976; Schürmann et al., 1976; Wolosiuk and Buchanan, 1977). Since then, it has been firmly established as the hallmark of chloroplast redox regulation (Buchanan, 1980; Buchanan et al., 2002). Although this pathway was characterized mainly based on *in vitro* experiments, it became evident that chloroplast redox regulation works dynamically in living plants; we directly observed that several redox-regulated proteins are indeed shifted from the oxidized to reduced forms when plants are exposed to light (Konno et al., 2012; Yoshida et al., 2014). These studies also let us notice an important aspect with regard to the diurnal redox response. Once reduced proteins were gradually reoxidized along with the decrease in light intensity at dusk, and finally returned to the fully oxidized state at night. It is reasonable to consider that some oxidizing force must be present at this light to dark transition phase; however, its identity or nature remains elusive. Some candidate factors for protein thiol oxidation have been suggested, including molecular oxygen, reactive oxygen species, oxidized glutathione, or oxidized Trx (Wolosiuk and Buchanan, 1977; Leegood and Walker, 1980; Scheibe and Anderson, 1981), but there has been little consensus on this issue. It has been therefore a long-standing gap in our understanding of chloroplast redox regulation.

We have recently come to a major turning point in this issue. Recent biochemical and physiological studies have identified the factors and pathways supporting the protein-oxidation process in chloroplasts (Ojeda et al., 2018; Vaseghi et al., 2018; Yoshida et al., 2018). These findings provide novel mechanistic insights into the regulation of photosynthesis; that is, the dark deactivation of chloroplast enzymes (Jacquot, 2018). Here we briefly highlight this breakthrough in the redox field.

## Genetic and Functional Diversity of Redox-Regulatory Factor Proteins in Chloroplasts

In addition to the light/dark response described above, the emergence of multiple Trx isoforms is considered as another key feature of the redox-regulatory system in plant chloroplasts. In the case of mammalian cells, the cytoplasm and mitochondria each contain only one Trx isoform (Spyrou et al., 1997). By contrast, plastidial Trx in a model plant *Arabidopsis thaliana* is encoded by as many as 10 nuclear genes, although some of these have limited expression in photosynthetic tissues (Belin et al., 2015; Okegawa and Motohashi, 2015; Yoshida et al., 2015). Furthermore, several proteins that have Trx-like amino acid sequence (CXXC) and can (potentially) mediate redox regulation are also found in chloroplasts. This multiplicity of redox-regulatory factor proteins is probably important for plants in flexibly regulating diverse chloroplast functions under changing light conditions.

**Figure 1:**
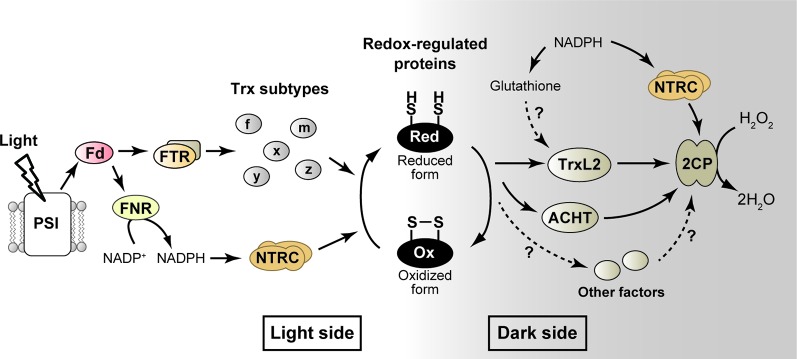
Simplified model for the redox-regulatory system in chloroplasts. The well-studied “light side” and newly emerging “dark side” are shown. Arrows indicate the flow of reducing power. See text for details. ACHT, atypical Cys His-rich thioredoxin; 2CP, 2-Cys peroxiredoxin; Fd, ferredoxin; FNR, ferredoxin-NADP^+^ reductase; FTR, ferredoxin-thioredoxin reductase; H_2_O_2_, hydrogen peroxide; NTRC, NADPH-thioredoxin reductase C; PSI, photosystem I; Trx, thioredoxin; TrxL2, thioredoxin-like2.

Chloroplast Trx isoforms are phylogenically categorized into five subtypes (*f*-, *m*-, *x*-, *y*-, and *z*-type, **Figure 1**) (Lemaire et al., 2007; Serrato et al., 2013). They have different molecular characteristics including the redox potential and protein surface charge, which in turn confers different functions on each of Trx subtypes (Collin et al., 2003; Yoshida et al., 2015; Lemaire et al., 2018). Another well-studied example of redox-regulatory factors is the NADPH-Trx reductase C (NTRC), a unique NTR with a joint Trx domain (Cejudo et al., 2012). NTRC shows largely different redox-regulatory properties from those of Trx subtypes (Serrato et al., 2004; Pérez-Ruiz et al., 2006; Michalska et al., 2009; Yoshida and Hisabori, 2016). It was recently suggested that NTRC plays a pivotal role in keeping redox balance of 2-Cys Prx (2CP) in chloroplasts, which is critical for plant growth (Pérez-Ruiz et al., 2017; see below for details). The functional diversity of redox-regulatory factors, mainly the five Trx subtypes and NTRC, has been described in detail in recent reviews (Geigenberger et al., 2017; Cejudo et al., 2019; Nikkanen and Rintamäki, 2019; Yoshida and Hisabori, 2019; Zaffagnini et al., 2019). In the following section, we focus on the factor directly involved in protein thiol oxidation in chloroplasts.

## Identification of Thioredoxin-Like2 as Protein-Oxidation Factor in Chloroplasts

Whereas multiple candidate proteins for the redox-regulatory factor have been identified from genomic and phylogenic studies (Meyer et al., 2006; Chibani et al., 2009), their molecular functions remain largely unclear. We biochemically characterized the function of one of such proteins, Trx-like2 (TrxL2) from *Arabidopsis*, allowing us to realize that this protein acts as a previously unidentified protein-oxidation factor in chloroplasts (**Figure 1**) (Yoshida et al., 2018). TrxL2 has an atypical Trx active site motif of WCRKC, and two isoforms (TrxL2.1 and TrxL2.2) are present in *Arabidopsis* chloroplasts. Both TrxL2 isoforms had the ability to transmit reducing power, but their midpoint redox potentials were higher (less negative) than those of typical Trx subtypes (e.g., TrxL2.1, –258 mV; TrxL2.2, –245 mV; Trx-*f*1, –321 mV; Trx-*m*1, –335 mV at pH 7.5) (Yoshida et al., 2015; Yoshida and Hisabori, 2017; Yoshida et al., 2018). Possibly in connection with this fact, TrxL2 did efficiently oxidize several redox-regulated proteins in chloroplasts, including FBPase, SBPase, and Rubisco activase (RCA). By contrast, TrxL2 failed to reduce these proteins; it can be regarded, therefore, that TrxL2 is capable of mediating redox reactions in the opposite direction of typical Trx.

Just before our publication on *Arabidopsis* TrxL2 (Yoshida et al., 2018), the crystal structure of poplar TrxL2 was determined (Chibani et al., 2018). This achievement is helpful to understand the reaction mechanisms of TrxL2 at a molecular level. Overall conformation of TrxL2 is similar to that of the canonical Trx (Jeng et al., 1994; Weichsel et al., 1996; Capitani et al., 2000). In the oxidized state, TrxL2 forms a disulfide bond in the WCRKC motif, suggesting that its basic catalytic mode (i.e., catalysis *via* dithiol/disulfide conversion) is shared between Trx and TrxL2. One different point is that, due to the precluding action of Arg and Lys residues in the WCRKC motif, TrxL2 is unlikely to receive reducing power from FTR. In accordance with this structure-based implication, it was actually shown that TrxL2 cannot be reduced by FTR (Chibani et al., 2012; Yoshida et al., 2018). In combination with the high redox potential, these two positively-charged amino acid residues appear largely responsible for the functional specificity of TrxL2.

It is still ambiguous whether TrxL2 has a crosstalk with redox-related low molecular weight compounds. Chibani et al. (2012) suggested that glutathione serves as a reductant for poplar TrxL2. They also solved the structure of poplar TrxL2 in the glutathione-adducted form (Chibani et al., 2018). However, the glutathione-binding site was another Cys residue located outside the redox-active WCRKC motif. Such a Cys residue is not ubiquitously conserved in plant orthologs; therefore, there may be little generality of the TrxL2-glutathione interaction. We also observed glutathione-dependent reduction of *Arabidopsis* TrxL2, but its efficiency was limited (Yoshida et al., 2018). Furthermore, there is no evidence for the association of glutathione with TrxL2 *in vivo*. Thus, further studies are needed to clarify this possibility (**Figure 1**).

## Involvement of 2-Cys Peroxiredoxin and Hydrogen Peroxide in Protein-Oxidation Process

Because *in vivo* protein levels of TrxL2 are not so high, TrxL2 must transfer reducing power to some molecule to keep an efficient protein-oxidation process (Yoshida et al., 2018). Screening for TrxL2-interacting partners allowed us to identify several chloroplast proteins that can physically bind to TrxL2. However, TrxL2 was unable to transfer reducing power to most of these proteins (but it could oxidize some of them). An exception was found in 2CP, which is the most abundant Prx in chloroplasts (Peltier et al., 2006) and serves to detoxify hydrogen peroxide (H_2_O_2_), a byproduct of photosynthesis (Asada, 1999). TrxL2 showed high activity for reducing 2CP, with an efficiency that greatly surpassed that of typical Trx (Yoshida et al., 2018; Yoshida et al., 2019). Given the high affinity of 2CP for H_2_O_2_ (Bernier-Villamor et al., 2004), it seems feasible that 2CP efficiently consumes the reducing power provided by TrxL2 through H_2_O_2_ detoxification. Using an *in vitro* reconstitution assay, we demonstrated that the TrxL2/2CP redox cascade acts as a novel system for oxidizing redox-regulated proteins and then draining reducing power to H_2_O_2_ (**Figure 1**). The *in vivo* function of this system was supported by the analyses of protein-oxidation dynamics in 2CP-deficient mutant plants in *Arabidopsis*; in these mutants, impaired oxidation of some chloroplast proteins (ATP synthase CF_1_-γ subunit, FBPase, SBPase, and RCA) and prolonged reduction of TrxL2 were evident during light to dark transitions (Yoshida et al., 2018).

Other research groups also suggested the involvement of 2CP and H_2_O_2_ in protein-oxidation process in chloroplasts (Ojeda et al., 2018; Vaseghi et al., 2018). Although different types of Prx reside in chloroplasts, a comprehensive study using several Prx-deficient mutants indicated that 2CP has a predominant role in protein-oxidation process (Ojeda et al., 2018). Notably, it was suggested that 2CP facilitates protein oxidation also in yeast (Veal et al., 2004) and human cells (Jarvis et al., 2012; Sobotta et al., 2015; Stöcker et al., 2018). However, their protein-oxidation pathways are clearly different from those in plant chloroplasts in terms that, in yeast and human cells, 2CP directly oxidizes target proteins.

It should be noted that NTRC may indirectly participate in the protein-oxidation process in chloroplasts by modulating the redox state of 2CP (**Figure 1**). Although several potential functions of NTRC have been suggested so far, its major role remains to be clarified. It has been demonstrated that NTRC exerts high 2CP-reducing activity *in vitro* (Pérez-Ruiz et al., 2006; Yoshida and Hisabori, 2016). In relation to this, Pérez-Ruiz et al. (2017) proposed the most convincing model for NTRC function *in vivo*. They found that the growth impairment phenotype of NTRC-deficient mutants is strikingly suppressed by a decrease in 2CP content. They concluded that NTRC controls the redox balance of 2CP, which is essential for optimal redox regulation in chloroplasts. It is therefore highly possible that the input of reducing power from NTRC and the upstream NADPH pool to 2CP affects protein-oxidation rate. The protein-oxidation response was partially impaired in NTRC-overexpressing plants, strongly supporting this idea (Ojeda et al., 2018).

## Dual Role of Trxl2/2CP Redox Cascade

Most of redox-regulated proteins in chloroplasts (e.g., CBC enzymes) are deactivated upon oxidation. Therefore, the TrxL2/2CP redox cascade plays a role in switching such proteins off in the dark. However, the TrxL2/2CP pathway has a critically different function for a certain protein, the glucose-6-phosphate dehydrogenase (G6PDH). G6PDH catalyzes the first committed step of the oxidative pentose phosphate pathway (OPPP), a primary pathway for supplying NADPH under non-photosynthetic conditions (Kruger and von Schaewen, 2003). G6PDH is a unique type of redox-regulated protein that is activated by oxidation (Scheibe et al., 1989; Née et al., 2009). Our recent biochemical study revealed that the TrxL2/2CP pathway acts as an oxidative activator of G6PDH (Yoshida et al., 2019). This finding suggests that the TrxL2/2CP pathway shifts chloroplast metabolism to the night mode by playing a dual role of downregulating CBC and upregulating OPPP.

## Implications of Protein-Oxidizing Network in Chloroplasts

In this mini review, we have mainly described the function of TrxL2 as a protein-oxidation factor based on our findings (Yoshida et al., 2018; Yoshida et al., 2019). It is highly plausible, however, that other chloroplast proteins with a Trx-like motif catalyze the protein thiol oxidation as well (**Figure 1**). In particular, the atypical Cys His-rich Trx (ACHT; alternative name, Trx-lilium) is a strong candidate for that process. Several ACHT isoforms from *Arabidopsis* have similar molecular features as TrxL2, as reflected by the high midpoint redox potential and the high efficiency of 2CP reduction (Dangoor et al., 2009; Yokochi et al., 2019). The protein-protein interaction of ACHT with 2CP was also shown by immunoprecipitation experiments (Dangoor et al., 2012; Cerveau et al., 2016). If ACHT serves as another protein-oxidation factor, the oxidizing targets and their difference from TrxL2 should be determined. In this regard, it is worth noting that one *Arabidopsis* isoform (ACHT4) may specifically oxidize the small subunit of ADP-glucose pyrophosphorylase, a redox-regulated protein involved in starch synthesis (Eliyahu et al., 2015). Interestingly, ACHT, unlike TrxL2 (exclusively localized to the stroma; Yoshida et al., 2018), is associated to the thylakoid membrane (Dangoor et al., 2012), implying the existence of functionally and spatially diverged protein-oxidizing networks in chloroplasts.

## Concluding Remarks

Recent findings of protein-oxidation machinery have led to a breakthrough in understanding the redox-regulatory system in chloroplasts. At the same time, they have opened new avenues of research to unveil its overall regulatory network (Jacquot, 2018). As discussed in the above section, our knowledge of protein-oxidation factors is still fragmentary. Further identification of such factors, followed by elucidation of their functional diversity and coordination, may allow us to draw more complicated regulatory model than currently expected. As another key challenge, spatiotemporal H_2_O_2_ dynamics during the protein oxidation should be clarified, as H_2_O_2_ is an ultimate oxidizing force for the protein-oxidation system. Finally, it remains to be answered how protein thiol oxidation in chloroplasts is important for plants. It has been hypothesized to be helpful for avoiding wasteful energy consumption during the night, but its physiological impact on plants remains experimentally unclarified. Future research addressing this issue will advance our understanding of plant strategies to survive in the dynamic environments of the field.

## Author Contributions

KY wrote the manuscript. YY and TH commented on the manuscript. All authors approved the manuscript.

## Funding

This work was supported by the Japan Society for the Promotion of Science (JSPS) KAKENHI Grant Number 16H06556 (to KY and TH) and 19H03241 (to KY), the Sumitomo Foundation (180881; to KY), and the Yoshinori Ohsumi Fund for Fundamental Research (to KY).

## Conflict of Interest

The authors declare that the research was conducted in the absence of any commercial of financial relationships that could be construed as a potential conflict of interest.
